# Frequency of disease-associated and other nuclear autoantibodies in patients of the German network for systemic scleroderma: correlation with characteristic clinical features

**DOI:** 10.1186/ar3495

**Published:** 2011-10-21

**Authors:** Rudolf Mierau, Pia Moinzadeh, Gabriela Riemekasten, Inga Melchers, Michael Meurer, Frank Reichenberger, Michael Buslau, Margitta Worm, Norbert Blank, Rüdiger Hein, Ulf Müller-Ladner, Annegret Kuhn, Cord Sunderkötter, Aaron Juche, Christiane Pfeiffer, Christoph Fiehn, Michael Sticherling, Percy Lehmann, Rudolf Stadler, Eckhard Schulze-Lohoff, Cornelia Seitz, Ivan Foeldvari, Thomas Krieg, Ekkehard Genth, Nicolas Hunzelmann

**Affiliations:** 1 Laboratory at Rheumaklinik Aachen, Hauptstrasse 21, Aachen, D-52066, Germany; 2 Department of Dermatology and Venerology, University of Cologne, Kerpener Strasse 62, Cologne, D-50937, Germany; 3 Department of Rheumatology and Clinical Immunology, Charité Universitätsmedizin, Humboldt University, Charitéplatz 1, Berlin, D-10117, Germany; 4 Clinical Research Unit for Rheumatology, University Medical Center Freiburg, Breisacher Strasse 66, Freiburg, D-79106, Germany; 5 Department of Dermatology, Dresden University Hospital, Fetscherstrasse 74, Dresden, D-01307, Germany; 6 Department of Internal Medicine II, University of Giessen, Klinikstrasse 33, Giessen, D-35392, Germany; 7 Clinic for Rheumatology, Schneckenhalde 13, Bad Säckingen, D-79713, Germany; 8 Reha-Rheinfelden, Salinenstrasse 98, Rheinfelden, CH-4310, Switzerland; 9 Department of Dermatology, Venerology and Allergology, Charité Universitätsmedizin, Humboldt University, Charitéplatz 1, Berlin, D-10117, Germany; 10 Department of Internal Medicine V, University of Heidelberg, Im Neuenheimer Feld 410, Heidelberg, D-69120, Germany; 11 Department of Dermatology and Allergology, Technical University of Munich, Biedersteiner Strasse 29, Munich, D-80802, Germany; 12 Department of Rheumatology and Clinical Immunology, Kerckhoff Clinic, Justus-Liebig University, Benekestrasse 2, Bad Nauheim, D-61231, Germany; 13 Department of Dermatology, Heinrich-Heine-University, Moorenstrasse 5, Düsseldorf, D-40225, Germany; 14 Department of Dermatology, University of Münster, Von-Esmarch-Strasse 58, Münster, D-48149, Germany; 15 Center of Rheumatology of Brandenburg, Johanniter Hospital in Fläming, Johanniterstrasse 1, Treuenbrietzen, D-14929, Germany; 16 Department of Dermatology and Allergology, University of Ulm, Maienweg 12, Ulm, D-89081, Germany; 17 Center for Rheumatology, Acura Hospital, Rotenbachtalstrasse 5, Baden-Baden, D-76530, Germany; 18 Department of Dermatology, Venerology and Allergology, University of Leipzig, Philipp-Rosenthal-Strasse 23, Leipzig, 04103, Germany; present address: Department of Dermatology, University Hospital Erlangen, Ulmenweg 18, Erlangen, D-91054, Germany; 19 Department of Dermatology and Allergology, Helios Klinikum, Heusnerstrasse 40, Wuppertal, D-42283, Germany; 20 Department of Dermatology, Johannes-Wesling-Klinik, Hans-Nolte-Strasse 1, Minden, D-32429, Germany; 21 Medical Clinic I, Hospital Cologne-Merheim, Ostmerheimer Strasse 200, Cologne, D-51109, Germany; present address: Medical Clinic 6, Marien-Hospital, Wanheimer Strasse 167a, Duisburg, D-47053, Germany; 22 Department of Dermatology, Venerology and Allergology, University of Würzburg, Josef-Schneider-Strasse 2, Würzburg, D-97080, Germany; present address: Department of Dermatology, Venerology and Allergology, Georg-August-University, Von-Siebold-Strasse 3, Göttingen, D-37075, Germany; 23 Hamburg Centre for Pediatric and Adolescence Rheumatology, Dehnhaide 120, Hamburg Eilbek, D-22081, Germany; 24 Rheumaklinik Aachen, Burtscheider Markt 24, Aachen, D-52066, Germany

**Keywords:** systemic sclerosis, scleroderma, autoantibodies, antinuclear antibodies

## Abstract

**Introduction:**

In the present study, we analysed in detail nuclear autoantibodies and their associations in systemic sclerosis (SSc) patients included in the German Network for Systemic Scleroderma Registry.

**Methods:**

Sera of 863 patients were analysed according to a standardised protocol including immunofluorescence, immunoprecipitation, line immunoassay and immunodiffusion.

**Results:**

Antinuclear antibodies (ANA) were detected in 94.2% of patients. In 81.6%, at least one of the autoantibodies highly associated with SSc or with overlap syndromes with scleroderma features was detected, that is, anti-centromere (35.9%) or anti-topoisomerase I (30.1%), followed in markedly lower frequency by antibodies to PM-Scl (4.9%), U1-ribonucleoprotein (U1-RNP) (4.8%), RNA polymerases (RNAPs) (3.8%), fibrillarin (1.4%), Ku (1.2%), aminoacyl-transfer RNA synthetases (0.5%), To (0.2%) and U11-RNP (0.1%). We found that the simultaneous presence of SSc-associated autoantibodies was rare (1.6%). Furthermore, additional autoantibodies were detected in 55.4% of the patients with SSc, of which anti-Ro/anti-La, anti-mitochondrial and anti-p25/p23 antibodies were most frequent. The coexistence of SSc-associated and other autoantibodies was common (43% of patients). SSc-associated autoantibodies disclosed characteristic associations with clinical features of patients, some of which were previously not acknowledged.

**Conclusions:**

This study shows that five autoantigens (that is, centromere, topoisomerase I, PM-Scl, U1-RNP and RNAP) detected more than 95% of the known SSc-associated antibody responses in ANA-positive SSc patients and characterise around 79% of all SSc patients in a central European cohort. These data confirm and extend previous data underlining the central role of the determination of ANAs in defining the diagnosis, subset allocation and prognosis of SSc patients.

## Introduction

Autoantibodies targeting characteristic nuclear antigens are one of the hallmarks of systemic sclerosis (SSc) [1-3]. The occurrence of different antinuclear antibodies (ANAs) is associated with distinct disease subtypes and with differences in disease severity, including extent of skin involvement, internal organ manifestation and prognosis. Although the current SSc criteria of the American College of Rheumatology [4] do not include the presence of ANAs, The detection of scleroderma-associated antibodies may be a valuable tool in the diagnosis of patients with very early SSc or only subtle symptoms [5,6]. For instance, in a recent study of patients with Raynaud's phenomenon, the presence of ANAs (adjusted HR = 5.67) and SSc-associated antibodies (HR = 4.7) was the strongest independent predictor of definite SSc [6]. Some of the autoantibodies in SSc are regarded as disease-specific and can be correlated with genetic, demographic, diagnostic, clinical and prognostic aspects of the disease [1,3]. Therefore, autoantibodies are pivotal tools in the diagnosis of SSc by helping clinicians make decisions whether to perform further, more detailed and efficient diagnostic procedures, as well as decisions addressing disease management.

For frequently occurring antibodies such as anti-centromere (ACA) and anti-topoisomerase I (ATA), reliable detection systems based on ELISA or other binding tests have been developed. Other antibodies (that is, to fibrillarin, RNA polymerases (RNAPs) and so on) are not identified by common test procedures, but rather by laboratories able to perform sophisticated procedures to confirm the results on the basis of more than one independent method. Even for the most common autoantibodies, the choice of the detection method used is critical to the sensitivity and specificity of the results and hence their diagnostic value [7].

Researchers in numerous studies have examined the presence of antibodies to single predefined antigens in SSc and their clinical associations, whereas many of the investigators who have comprehensively examined large SSc patient cohorts have often restricted their autoantibody analyses to the most common SSc antibodies, ACA and ATA [8-12], or analysed only a few additional antibodies [13-17]. The aim of this study was therefore to characterise all known non-organ-specific, SSc-associated autoantibodies, as well as other, potentially new nuclear autoantibodies by using a standardised protocol in the large SSc patient cohort included in the German Network for Systemic Scleroderma Registry, and to correlate these findings with the clinical characteristics of these patients.

## Materials and methods

### Patients

Serum samples from 863 consecutive patients included in the German Network for Systemic Scleroderma Registry between 2004 and 2007 from 23 different clinical centres were analysed. Patient data are gathered and registered using a consensual registration form and reference documents with item definitions and recommendations for organ-specific diagnostic procedures as previously described [18,19]. Of the patients included, 82.9% were female, their mean age ± SD was 58.0 ± 13.4 years (median = 60 years, range 12 to 93). The patients' age at disease onset ranged from 3 to 87 years (median = 49 years, mean = 47.7 ± 14.2).

The registry defines five subsets, that is, limited cutaneous and diffuse cutaneous SSc [20], overlap syndrome [21,22], systemic sclerosis sine scleroderma [23,24] and undifferentiated connective tissue disease with features of scleroderma [25,26], as recently described [18]. The latter subset corresponds largely to the subgroup 'early SSc' as described by LeRoy and Medsger [5] but may also include patients who will never develop definite SSc. The study, including the patients' informed consent regarding data storage, was approved by the lead Ethics committee of the Cologne University Hospital and by the respective ethics committees of the contributing centres.

### Autoantibody analysis

**Figure 1:**
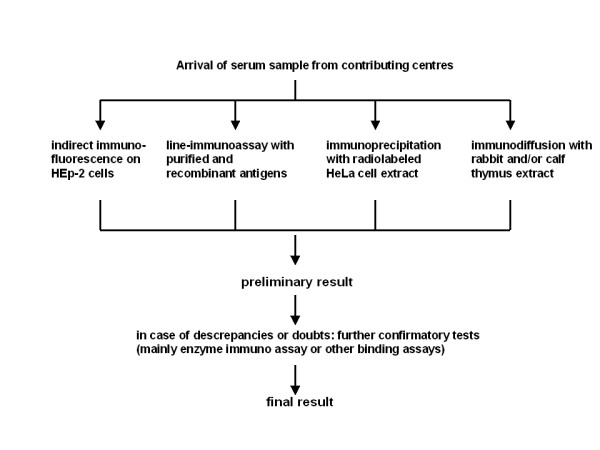
**Protocol for serological analysis of systemic sclerosis patient sera**.

To detect SSc-associated autoantibodies in a comprehensive way, we used the search strategy commonly performed in diagnostic procedures for connective tissue diseases based on a HEp-2 cell immunofluorescence assay followed by tests using cellular extracts and/or recombinant antigens. This strategy is focused on circulating antibodies against non-organ-specific cellular autoantigens. Cell- or tissue-specific autoantibodies, which have also been described in scleroderma patients [3], were not included in the analytical protocol. At least one serum draw from each patient (*N *= 863) was analysed for circulating autoantibodies by a predefined protocol (Figure 1) with at least four assay systems performed in a single laboratory by a single group of technologists.

Indirect immunofluorescence using fluorescein isothiocyanate-conjugated goat anti-human immunoglobulin G was performed as a screening method for the detection of ANAs on HEp-2 cells (HEp-20-10; Euroimmun, Lübeck, Germany) seeded onto a microscope slide [27]. Titres of at least 1:80 dilution were regarded as positive. Different nuclear and cytoplasmic fluorescence patterns were documented.

A line immunoassay (EUROLINE ANA Profile 3; Euroimmun) was performed according to the manufacturer's instructions. This assay is able to detect, by binding to recombinant or purified antigens, the following autoantibodies: U1-ribonucleoprotein (U1-RNP), Sm, Ro60, Ro52, La (SS-B), Scl-70, PM-Scl, centromere protein B (CENP-B), proliferating cell nuclear antigen (PCNA), double-stranded DNA (dsDNA), nucleosomes, histones, ribosomal P proteins and the mitochondrial M2 antigen.

Immunoprecipitation (IP) of radiolabelled HeLa cell extract was performed as described [28,29] with slight modifications. In brief, HeLa S3 cells in suspension culture in methionine-deficient RPMI medium with 10% dialyzed foetal bovine serum were incubated with ^35^S methionine (to a final activity of 0.3 MBq/ml) overnight. They were (1) washed in Tris-buffered saline and (2) lysed by resuspension in IP buffer (10 mM Tris·HCl, pH 8.0, with 500 mM NaCl, 0.1% Igepal (Sigma, Munich, Germany) and 2 mM phenylmethylsulfonyl fluoride) and sonication on ice. IP was performed by incubation of patient sera (10 μl) with protein A Sepharose beads (2 mg in 500 μl; Sigma) for two hours, three short washing steps, end-over-end rotation with the radiolabelled cell extract overnight, five washing steps, separation of the precipitates on 5% to 20% gradient SDS-PAGE gels and subsequent autoradiography for six to ten days. Bands typical of autoantibodies were identified according to their apparent molecular weight and comigration with bands produced by reference sera with known autoantibody specificity. After we completed this procedure, the autoantibodies to the following antigens were routinely detectable: topoisomerase I (Scl-70), RNAPs (I and/or III), Ku, fibrillarin, To, NOR-90, Pl-7, Pl-12, EJ, OJ, KS, Mi-2, signal recognition particle, ribonucleoprotein (usually U1-RNP), SL, PCNA, ribosomal P proteins and p25/p23, also known as 'anti-chromo' [30,31]. In our hands, the detection of antibodies to Ro, La, PM-Scl, Jo-1, centromere antigens and the mitochondrial M2 antigen was unreliable by this method. Bands of unknown nature were registered and entered into the database of autoantibody results.

Immunodiffusion (ID) was performed in Agarose gels with rabbit and/or calf thymus extract (Pel-Freez Biologicals, Rogers, AR, USA) as described previously [32]. Autoantibodies were identified by the identity of precipitation lines with patient sera compared with monospecific prototype sera with known autoantibody specificity. The use of prototype serum was guided by results of the immunofluorescence pattern on HEp-2 cells. The following autoantibodies were detectable: topoisomerase 1 (Scl-70), PM-Scl, Ku, SL, Jo-1, Pl-7, U1-RNP, Sm and La (SS-B). Precipitation bands that did not merge with any of the prototype sera were registered as unknown autoantibodies and entered into the database of autoantibody results.

In selected sera, confirmatory assays using recombinant or synthetic antigens (see Table 1 Antibody detection criteria) were performed. More than one serum sample was available from 213 patients (from 2 to 25 samples). At least one serum sample per patient was tested with the whole protocol described in Figure 1, whereas in most cases the follow-up sera drawn were at least partially characterised with, for example, the HEp-2 cell test. The criteria for classifying sera as positive for autoantibodies are listed in Table 1 together with additional serological results commonly found and helpful to identify the antibodies.

**Table 1:** Methodological criteria for assignment of autoantibodies

Autoantibody against	Findings classifying patients as antibody-positive	Usual additional findings
Centromere	Centromeric immunofluorescence pattern on HEp-2 cells (308 of 310 were positive) or a CENP-B band in line assay (309 of 310 positive)	
Topoisomerase I	At least two of three findings: Scl-70-positive signal in line assay (258 of 260 positive), a band comigrating with a prototype band in IP (all positive), a line of identity in ID with the Scl-70 prototype serum (244 of 260 positive)	Typical HEp-2 cell immunofluorescence pattern: fine granular karyoplasmic, weakly nucleolar, metaphase chromosome-positive
RNA polymerases	Characteristic IP pattern comigrating with the pattern of a prototype serum mainly consisting of four bands: Ia, Ib, IIIa and IIIb [58]	Confirmation by ELISA in 32 of 32 cases with the immunodominant epitope of RNA polymerase III subunit RPC155 according to Kuwana *et al. *[71], provided by Matritec, Freiburg, Germany. ANA immunofluorescence on HEp-2 cells was predominantly fine granular only sometimes (five cases) in addition nucleolar [72].
Fibrillarin	An IP band (approximately 34 kDa) comigrating with a prototype serum band, plus a nucleolar immunofluorescence pattern on HEp-2 cells	Confirmation by investigational ELISA kindly provided by Euroimmun, Lübeck, Germany, positive in 11 and borderline in 1 of the 12 cases
To	An IP band of approximately 40 kDa plus a nucleolar immunofluorescence pattern on HEp-2 cells; confirmation by immunoblot analysis with recombinant To antigen kindly supplied by Dr M Blüthner, Labor Seelig, Karlsruhe, Germany	
PM-Scl	A line of identity in ID with a PM-Scl prototype serum (41 of 42 cases) and/or positive result of ELISA with the synthetic peptide PM-1α [73] (Dr Fooke Laboratorien GmbH, Neuss, Germany) (12 of 13 cases)	Positive reaction in 37 of 41 cases for PM-Scl by line assay. ANA immunofluorescence on HEp-2 cells usually was nucleolar plus fine granular karyoplasmic.
Ku	Two prominent IP bands at about 70 and 80 kDa comigrating with prototype bands	In 3 of 10 cases, a line identical to a Ku prototype band in ID. ANA immunofluorescence was finely granular, usually at a high titre.
U1-RNP	A positive signal for RNP/Sm in line assay, with or without a positive signal for Sm, plus a typical IP pattern consisting of at least antigen A (about 33 kDa), antigen B/B' (about 28/29 kDa) and antigen C (about 22 kDa)	In 37 of 41 cases, a line of identity with a U1-RNP prototype in ID. ANA pattern on HEp-2 cells usually was coarsely speckled.
Sm	A positive signal for RNP/Sm as well as for Sm in line assay	In two of four cases, a band identical to a Sm prototype in ID with ribonuclease-digested calf thymus extract. IP and immunofluorescence patterns were similar to those found for anti-U1-RNP.
Jo-1	A positive signal for Jo-1 in line assay plus a band identical to a Jo-1 prototype band in ID	Immunofluorescence on HEp-2 cells was inconsistent.
Pl-7	IP band of about 80 kDa comigrating with prototype band plus a band identical to a Pl-7 prototype band in ID	Cytoplasmic immunofluorescence on HEp-2 cells
OJ	Typical triplet band in IP comigrating with prototype bands	Cytoplasmic immunofluorescence on HEp-2 cells
U11-RNP [74,75]	An RNP-like IP pattern and coarsely speckled ANA immunofluorescence, without any U1-RNP signals in line assay and ID; U11-RNP specificity detected by C Will and R Lührmann, Marburg, Germany	
p25/p23 [76,77]	Doublet IP bands of about 25 and 23 kDa, with the 25 kDa band comigrating with the precipitate of rabbit anti-p25 kindly provided by E Chan, Gainesville, FL, USA	HEp-2 cell immunofluorescence pattern was always centromeric because anti-p25/p23 was exclusively found together with anticentromere.
SL	A band identical to the SL prototype band in ID plus an IP band at about 31 kDa comigrating with the precipitate of the SL reference serum	HEp-2 cell immunofluorescence pattern was fine granular, but in this study often was masked because of other coexisting antibodies
NOR-90	Doublet IP bands at about 90 kDa comigrating with the precipitate of a NOR-90 reference serum [78]	The nucleolar immunofluorescence pattern expected on HEp-2 cells was hard to detect in the sera examined in this study, because NOR-90 antibodies in all cases coincided with other autoantibodies visible on HEp-2 cells.
Mitochondrial M2	AMA M2-positive signal by line assay (40 of 41 positive) and/or AMA typical cytoplasmic immunofluorescence on HEp-2 cells and/or rat kidney sections (27 of 41 positive)	In IP, a band of around 70 kDa was present in 36 of 41 cases.
Sp100	Multiple nuclear dot pattern on HEp-2 cells [79] plus Sp100 signal in the line assay HUMAN IMTEC-Liver Line Immunoassay (HUMAN Diagnostics GmbH, Wiesbaden, Germany)	
Ro52	Ro52-positive signal by line assay	
Ro60	Ro60-positive signal by line assay	
La	La-positive signal by line assay	

AMA = antimitochondrial antibodies; ANA = antinuclear antibodies; CENP-B = centromere protein B; ID = immunodiffusion; IP = immunoprecipitation; PM-Scl = polymyositis and scleroderma; RNAP = RNA polymerase; RNP = ribonucleoprotein;.

In addition to the autoantibodies defined in Table 1 other circulating autoantibodies detected by at least one of the above-mentioned procedures, either known (for example, anti-histone, anti-dsDNA) or unknown (for example, either unidentified bands in IP or ID or antinuclear or anticytoplasmic antibodies on HEp-2 cells without subsequent identification), were registered. Sera which were negative for ANAs in immunofluorescence on HEp-2 cells but exhibited cytoplasmic fluorescence in that assay and/or a positive signal in any of the other assays were grouped together as ANA-negative. Sera without any positive signal, neither defined nor undefined, in all four assay systems described above were listed as autoantibody-negative.

### Statistics

The data were analysed using Microsoft Excel (Microsoft Corp, Redmond, WA, USA) and SPSS version 14.0 software (SPSS, Inc, Chicago, IL, USA) for tabular and graphic representation. Statistical evaluation was performed using contingency table tests with the help of GraphPad Prism version 3.02 software (GraphPad, La Jolla, CA, USA). We calculated OR and 95% CI data. *P*-values were calculated using Fisher's exact test. When multiple tests were performed, *P*-values below 0.005 were recorded without performing strict Bonferroni correction. For most variables, less than 5% of data were missing. Quantitative data (erythrocyte sedimentation rate (ESR), age at disease onset and Rodnan skin score), depending on the presence or absence of different autoantibodies, were analysed using the Mann-Whitney rank-sum test.

## Results and discussion

Of the 863 SSc patients studied, 513 (59.4%) were classified as having limited disease and 173 (20.1%) we classified as having diffuse cutaneous disease. Another 108 patients (12.5%) had a scleroderma overlap syndrome, 64 (7.4%) had undifferentiated connective tissue disease with scleroderma features and 5 (0.6%) had systemic sclerosis sine scleroderma.

The frequency of autoantibodies detected in these patients is shown in Table 2. Overall, ANAs were detected in 94.2% of patients. This frequency was similar to data previously published [12,14,15,33,34] in which ANA frequencies reported were between 85% and 99%. Among our patients with ANAs, 86.6% (704 of 813) had autoantibodies known to be highly associated with SSc, and among these latter patients, 96.4% (679 of 704) had antibodies that detected five antigens: centromere, topoisomerase I, PM-Scl, U1-RNP and RNAPs.

**Table 2:** Prevalence of autoantibodies in 863 scleroderma patients

Autoantibodies	Patients, *n *(%)
Positive for antinuclear antibodies	813 (94.2)
Antibodies highly associated with SSc or scleroderma overlap syndromes	704 (81.6)
Anti-centromere	310 (35.9)
Anti-topoisomerase I	260 (30.1)
Anti-PM-Scl	42 (4.9)
Anti-U1-RNP	41 (4.8)
Anti-RNA polymerase	33 (3.8)
Anti-fibrillarin	12 (1.4)
Anti-To	2 (0.2)
Anti-Ku	10 (1.2)
Anti-Jo-1/-Pl-7/-OJ	4 (0.5)
Anti-U11-RNP	1 (0.1)
Other autoantibodies	
Anti-Ro and/or anti-La	206 (23.9)
Anti-Ro52	187 (21.7)
Anti-Ro60	59 (6.8)
Anti-La	16 (1.9)
Anti-mitochondrial M2	41 (4.8)
Anti-p25/p23	28 (3.2)
Anti-NOR-90	6 (0.7)
Anti-SL	9 (1.0)
Anti-Sm	4 (0.5)
Anti-Sp100	4 (0.5)
Other (known or unknown)	363 (42.1)
Negative for all highly SSc-associated antibodies	159 (18.4)
Negative for antinuclear antibodies	50 (5.8)
Autoantibody-negative by all criteria used	38 (4.4)

ANA = antinuclear antibodies; SSc = systemic sclerosis.

A coincidence of SSc-associated autoantibodies (Table 3) was rare, being detected in only 1.6% patients (11 of 704). The presence of a SSc-associated antibody without any other autoantibody detectable by the methods used was found at varying frequencies, being highest for anti-PM-Scl (73.8%) and lower for, for example, anti-centromere (33.9%) and anti-fibrillarin (33.3%) (see Table 3). That SSc-associated autoantibodies are largely mutually exclusive is well-known [14,15,35]. Coincidences in individual patients do occur but are rare [33,36,37]. Our study shows that this statement holds true even if all known non-organ-specific, SSc-associated autoantibodies are sought using a rigorous protocol in all patients. On the other hand, additional (mainly not SSc-specific) autoantibodies are common and were detected in about 53% in our patient cohort with SSc-associated antibodies (and in 55.4% of all of our patients). In 14.0% of patients (*n *= 121), none of the above-mentioned SSc-associated but other (defined or undefined) autoantibodies were found, whereas in 4.4% no autoantibodies at all were detected by the methods used. Defined autoantibodies not regarded as SSc-specific, such as anti-Ro/La, anti-NOR-90 or AMAs rarely occurred without the evidence of SSc-associated autoantibodies (Table 3). Antibodies to p25/p23 were detected exclusively in conjunction with ACA.

**Table 3:** Coincidence* of autoantibodies in 863 individual systemic sclerosis patients

Antibodies	ACA	ATA	Anti-PM-Scl	Anti-U1-RNP	Anti-RNAP	Anti-fibrilla-rin	Anti-To	Anti-Ku	Anti-Jo-1/Pl-7/OJ	Anti-U11-RNP	Anti-Ro52	Anti-Ro60	Anti-La	AMA	Anti-p25/p23	Anti-NOR-90	Anti-SL	Anti-Sm	Anti-Sp100	Other
ACA		1	0	1	0	0	0	0	0	0	92	11	1	31	28	4	2	0	3	144
ATA			1	2	0	0	0	4	0	0	36	26	6	4	0	1	4	1	0	77
Anti-PM-Scl				0	0	0	0	1	0	0	6	2	0	0	0	0	0	0	0	4
Anti-U1-RNP					1	0	0	0	0	0	11	6	1	2	0	1	0	4	0	11
Anti-RNAP						0	0	0	0	0	5	1	1	1	0	0	1	0	0	5
Anti-fibrillarin							0	0	0	0	0	0	0	1	0	0	0	0	0	8
Anti-To								0	0	0	1	1	0	0	0	0	0	0	0	2
Anti-Ku									0	0	2	2	0	0	0	0	1	0	0	2
Anti-Jo-1/Pl-7/OJ										0	3	0	0	0	0	0	0	0	0	1
Anti-U11-RNP											0	0	0	0	0	0	0	0	0	0
Anti-Ro52												41	15	14	11	2	5	2	2	97
Anti-Ro60													14	6	0	0	0	1	1	33
Anti-La														1	0	0	0	1	0	8
AMA															2	0	1	1	2	29
Anti-p25/p23																1	1	0	1	18
Anti-NOR-90																	0	1	0	4
Anti-SL																		0	0	3
Anti-Sm																			0	0
Anti-Sp100																				2
Other																				
Isolated^a^	104	153	31	19	23	4	0	4	1	1	2	1	0	0	0	0	1	0	0	79
Total^b^	310	260	42	41	33	12	2	10	4	1	187	59	16	41	28	6	9	4	4	363

*Number of patients with co-occurrences of autoantibodies. ^a^Isolated: presence without coincidence of any other autoantibody by all detection methods used. ^b^Total number of individuals with the respective autoantibody; these numbers mostly are smaller than the sum of all co-occurrences plus the 'isolated' individuals listed because of some triple, quadruple and higher-order coincidences. ACA = anti-centromere antibodies; AMA = antimitochondrial antibodies; ATA = anti-topoisomerase I antibodies;; RNAP = RNA polymerase; RNP = ribonucleoprotein.

From 213 patients, more than one serum sample was available (from 2 to 25 samples). In the majority (86.4%) of cases, the results of follow-up testing remained essentially the same and differed in ANA titre by up to only two titre steps. In 11.3% (24 patients), ANA titre changes exceeded two steps (up to eight steps), in two cases ANAs turned negative, in one case the ANA pattern changed from finely granular to nucleolar and in only two cases new, additional typical SSc autoantibodies emerged.

The detection of antibodies in different disease subsets is shown in Table 4. It is obvious that ACA and ATA are not exclusive to either the limited or the diffuse subset. In patients with overlap syndrome, anti-U1-RNP, anti-PM-Scl and anti-synthetase antibodies are characteristic.

**Table 4:** Autoantibodies in different disease subsets in 863 individual systemic sclerosis patients

	Limited (*N *= 513)		Diffuse (*N *= 173)		Overlap (*N *= 108)		Undifferentiated (*N *= 64)
	
Antibodies	*n *(%)	OR^a ^(*P*-value)	*n *(%)	OR (*P*-value)	*n *(%)	OR (*P*-value)	*n *(%)
ACA	**253 (49.3)**	**5.00 (*P *< 0.0001)**	12 (6.9)		16 (14.8)		28 (43.8)
ATA	141 (27.5)		**98 (56.6)**	**4.26 (*P *< 0.0001)**	11 (10.2)		9 (14.1)
Anti-RNAP	14 (2.7)		**14 (8.1)**	**3.11 (*P *= 0.0029)**	2 (1.9)		2 (3.1)
Anti-U1-RNP	7 (1.4)		0 (0.0)		**31 (28.7)**	**30.00 (*P *< 0.0001)**	2 (3.1)
Anti-PM-Scl	16 (3.1)		2 (1.2)		**22 (20.4)**	**9.40 (*P *< 0.0001)**	2 (3.1)
Anti-fibrillarin	3 (0.6)		**8 (4.6)**	**8.32 (*P *= 0.0005)**	1 (0.9)		0 (0.0)
Anti-To	1 (0.2)		0 (0)		1 (0.9)		0 (0.0)
Anti-Jo-1/Pl-7/OJ	0 (0.0)		0 (0)		**4 (3.7)**	**65.07 (*P *= 0.0002)**	0 (0.0)
Anti-U11-RNP	0 (0.0)		1 (0.6)		0 (0.0)		0 (0.0)
Anti-Ku	5 (1.0)		1 (0.6)		3 (2.8)		1 (1.6)
Anti-SL	5 (1.0)		3 (1.7)		1 (0.9)		0 (0.0)
Anti-Sm	0 (0.0)		0 (0.0)		3 (2.8)	21.54 (*P *= 0.0069)	0 (0.0)
Anti-NOR-90	5 (1.0)		0 (0.0)		1 (0.9)		0 (0.0)
AMA	28 (5.5)		4 (2.3)		4 (3.7)		5 (7.8)
Anti-Sp100	3 (0.6)		0 (0.0)		0 (0.0)		1 (1.6)
Anti-Ro52	125 (24.4)	1.50 (*P *= 0.023)	20 (11.6)		27 (25.0)		15 (23.4)
Anti-Ro60	28 (5.4)		14 (8.1)		13 (12.0)	2.11 (*P *= 0.0382)	4 (6.3)
Anti-La	9 (1.8)		3 (1.7)		1 (0.9)		3 (4.7)
Anti-p25/p23	**24 (4.5)**	**4.25 (*P *= 0.0031)**	0 (0.0)		2 (1.9)		2 (3.1)
Other	209 (40.7)		74 (42.8)		44 (40.7)		34 (53.1)
ANA-negative	32 (6.2)		6 (3.5)		5 (4.6)		7 (10.9)

ACA, anti-centromere antibodies; AMA, antimitochondrial antibodies; ANA, antinuclear antibodies; ATA, anti-topoisomerase I antibodies; RNAP, RNA polymerase;. ^a^OR for antibody in that subset compared with all other subsets. Only significant positive associations are documented by OR, and those with *P*-values < 0.005 are printed in bold.

The correlation of demographic features or signs and symptoms of SSc with the presence or absence of defined autoantibodies was investigated by contingency table analysis. The frequencies of these features and their positive or negative correlations with specific autoantibodies are listed in Table 5. For the purpose of clarity, only those comparisons that led to *P*-values below 0.05 derived by Fisher's exact test are shown. Significance was calculated without correction of *P*-values for multiple comparisons, because not all variables used were independent; however, we are aware of the fact that some of the weak associations listed in Table 5 might have arisen by chance due to the high number of comparisons made. Therefore, we focused on those differences calculated that were highly significant (*P *< 0.005; OR printed in bold in Table 5).

**Table 5:** Correlations of clinical features with SSc associated autoantibodies

	ACA (310)	ATA (260)	Anti-PM-Scl (42)	Anti-U1-RNP (41)	Anti-RNA -P (33)	Anti-Fibrillarin (12)	Anti-Ku (10)	Anti-Ro52 (187)	Anti-Ro60 (59)	Anti-La (16)	AMA (41)	Anti-p25/23 (28)	ANA-negative (50)	no SSc associated ab's (161)
male sex 148 (17.1%)	**17** **0.19 (0.11 to 0.32)** P < 0.0001	**64** **2.02 (1.4 to 2.91)** p = 0.0002	6	9	8	4	2	23 0.62 (0.38 to 0.997 p = 0.049	12	2	1	1	10	**41** **1.90 (1.261 to 2.863)** p = 0,0035
age at disease onset < 50 y 399/781 (51.1%)	**115** **0.57 (0.43 to 0.77)** p = 0.0003	135 1.39 (1.02 to 1.88) p = 0.0431	22	**29** **2.92 (1.40 to 6.07)** p = 0.0029	13	8	4	77	31	10	20	8	18	73
Rodnan skin score > 10 294/750 (39.2%)	**58** **0.27 (0.19 to 0.38)** P < 0.0001	**132** **3.10 (2.24 to 4.27)** P < 0.0001	14	9	**18** **3.24 (1.44 to 7.31)** p = 0.0042	7	1	58	23	6	10	11	14	56
Raynaud's phenomenon 819 (94.9%)	301 2.26 (1.07 to 4.77) p = 0.0349	252	37	41	32	12	10	184 3.96 (1.2 to 12.94 p = 0.0133	58	16	41	28	43 0.29 (0.12 to 0.70) p = 0.0104	**140** **0.23 (0.12 to 0.42)** P < 0.0001
Digital ulcers 216/840 (25.7%)	**55** **0.50 (0.36 to 0.71)** P < 0.0001	**106** **3.18 (2.30 to 4.41)** P < 0.0001	9	11	7	4	1	48	19	6	13	6	4 0.26 (0.09 to 0.76) p = 0.0076	28 0.60 (0.39 to 0.94) p = 0.024
Pulmonary hypertension 126 (14.6%)	57 1.58 (1.08 to 2.32) p = 0.0208	36	2	9	4	1	0	31	14	2	2	7	2 0.23 (0.06 to 0.97) p = 0.0232	17
Pulmonary fibrosis 287 (33.3%)	**39** **0.18 (0.12 to 0.26)** P < 0.0001	**151** **4.76 (3.48 to 6.50)** P < 0.0001	16	11	7	2	6	69	**30** **2.20 (1.29 to 3.75** p = 0.0040	9	6 0.33 (0.14 to 0.79) p = 0.01	4 0.33 (0.11 to 0.95) p = 0.0393	11	55
Lung restrictive defect 218/833 (26.2%)	**41** **0.31 (0.21 to 0.45)** P < 0.0001	**104** **2.96 (2.14 to 4.09)** P < 0.0001	9	7	8	3	4	45	19	4	8	4	10	34
Esophageal involvement 535 (62.0%)	198	175 1.39 (1.02 to 1.89) p = 0.039	**14** **0.29 (0.152 to 0.56)** p = 0.0001	29	20	7	6	120	39	13	24	21	27	87 0.67 (0.47 to 0.94) p = 0.0243
Proteinuria 90/830 (10.8%)	23 0.56 (0.34 to 0.92) p = 0.0207	34	4	4	4	1	1	15	9	2	3	1	5	19
Cardiac involvement 114 (13.2%)	**27** **0.51 (0.32 to 0.81)** p = 0.0033	45 1.62 (1.08 to 2.44) p = 0.0216	5	7	5	1	2	20	9	1	4	1	5	22
Musculoskeletal involvement 421/852 (49.4%)	**131** **0.64 (0.48 to 0.85)** p = 0.0022	130	20	28 2.49 (1.25 to 4.96) p = 0.009	17	10 5.22 (1.14 to 23.97) p = 0.0202	6	80 0.71 (0.51 to 0.99) p = 0.0468	32	12 3.13 (1.002 to 9.79) p = 0.0447	17	9	25	79
Synovitis 157/837 (18.8%)	**35** **0.43 (0.29 to 0.65)** P < 0.0001	**62** **1.71 (1.19 to 2.45)** p = 0.0049	8	13 2.27 (1.14 to 4.53) p = 0.0326	8	2	2	32	15	5	2 0.21 (0.05 to 0.89) p = 0.0216	2	10	29
Joint contractures 253/840 (30.1%)	**54** **0.36 (0.25 to 0.50)** P < 0.0001	**107** **2.26 (1.65 to 3.08)** P < 0.0001	9	9	10	6	2	49	23	6	9	9	20 1.93 (1.05 to 3.54) p = 0.0436	56
Tendon friction rubs 88/840 (10.5%)	**14** **0.30 (0.16 to 0.53)** P < 0.0001	32	2	5	6	3	1	21	5	2	2	0	6	25 1.95 (1.18 to 3.22) p = 0.0122
CK elevation 74/835 (8.9%)	**14** **0.37 (0.21 to 0.68)** p = 0.0009	20	**19** **3.56 (1.67 to 7.57)** p = 0.0023	5	6 2.60 (1.03 to 6.55) p = 0.0485	0	3 5.32 (1.30 to 21.72) p = 0.038	21	8	2	1	0	6	16
Sicca syndrome 366/858 (42.7%)	150 1.44 (1.19 to 6.61) p = 0.0119	98	14	18	12	5	4	96 1.57 (1.13 to 2.17) p = 0.0075	33 1.85 (1.08 to 3.17) p = 0.0275	12 4.14 (1.32 to 12.93) p = 0.0102	19	**20** **3.50 (1.52 to 8.03)** p = 0.0029	23	66
Mouth involvement 223/829 (26.9%)	**64** **0.61 (0.44 to 0.85)** p = 0.0034	**93** **2.1 (1.53 to 2.93)** P < 0.0001	10	7	10	3	4	49	18	6	11	6	12	34
ESR > 25 mm/h 199/741 (26.9%)	58 0.70 (0.49 to 0.99) p = 0.046	75 1.55 (1.10 to 2.19) p = 0.015	4 0.33 (0.11 to 0.94) p = 0.0325	14	5	0	0	**60** **1.76 (1.21 to 2.54)** p = 0.0039	**28** **3.39 (1.92 to 5.97)** P < 0.0001	9 3.62 (1.33 to 9.86) p = 0.0447	10	5	15	42

ACA, anti-centromere antibodies; AMA, antimitochondrial antibodies; ANA, antinuclear antibodies; ATA, anti-topoisomerase I antibodies; CK, creatine kinase; ESR, erythrocyte sedimentation rate; RNAP = RNA polymerase; RNP = ribonucleoprotein; SSc, systemic sclerosis. Dichotomous variables are expressed as raw numbers, OR (95% CI) and *P *values.

Patients with ACA represented 35.9% of all SSc patients and 38.1% of ANA-positive SSc patients. In accordance with previous reports [8,9,12,15,33,36,38,39], these patients were less often male, were older at disease onset and had a more limited extension of cutaneous involvement, as documented by a much lower OR for a Rodnan skin score (RSS) above 10 (Table 5) and by a very significantly lower mean RSS (Table 6). They had less involvement of internal organs (pulmonary fibrosis, cardiac, musculoskeletal and oral involvement), with the exception of pulmonary hypertension. An association of ACA with pulmonary hypertension has been observed in several previous reports [2,12,33,40], but not all of them [13,36,38,39]. Digital ulcers in our patients with ACA were less common compared to American patients [2] and more similar to European [9,12,38] and Japanese patients [34]. Nevertheless, the presence of ACA does not by any means preclude digital ulcers. To date no marker constellation allows the identification of patients prone to this complication [41-43]. ACAs are frequently associated with other antibodies, such as anti-Ro [44-46], anti-mitochondrial (M2) [15,44,47,48] and anti-p25/p23 [31,49,50] antibodies. These associations were confirmed by this study. The reasons for this frequent co-occurrence are unknown. There is no known antigenic relationship between the individual targets of the antibodies. Probably the (unknown) aetiopathogenetic pathways marked by these antibodies have common components, including common genetic predispositions.

**Table 6:** Correlations of clinical features with systemic sclerosis-associated autoantibodies

Quantitative traits	Clinical data		
Age at disease onset	*n*	Mean ± SD (years)	P*
Total	781	47.7 (14.2)	
Anti-centromere	273	51.3 (12.5)	< 0.0001
Anti-topoisomerase I	238	46.0 (14.0)	0.0076
Anti-fibrillarin	12	38.8 (16.0)	0.0404
Anti-U1-RNP	39	38.2 (15.0)	< 0.0001
Anti-La	15	37.9 (18.1)	0.0431
Autoantibody-negative	36	52.9 (14.7)	0.0205
Rodnan skin score	*n*	Mean score ± SD	*P*
Total	750	10.2 (9.4)	
Anti-centromere	275	6.4 (6.0)	< 0.0001
Anti-topoisomerase I	227	14.1 (9.7)	< 0.0001
Anti-RNA polymerase	27	15.7 (11.7)	0.0091
Anti-fibrillarin	10	21.2 (15.0)	0.0108
Anti-U1-RNP	35	6.9 (9.2)	0.0053
Erythrocyte sedimentation rate	*n*	Mean ± SD (mm/hour)	*P*
Total	741	19.46 (16.7)	
Anti-topoisomerase I	227	22.95 (19.1)	0.0002
Anti-PM-Scl	36	12.19 (9.4)	0.0014
Anti-Ro52	167	22.05 (18.8)	0.0374
Anti-Ro60	53	28.47 (19.6)	< 0.0001
Anti-La	16	28.56 (20.1)	0.0447

PM-Scl = polymyositis and scleroderma; RNP = ribonucleoprotein; SSc, systemic sclerosis. *P-value calculated by Mann-Whitney rank-sum test for comparison of antibody-positive vs antibody-negative patients.

The prevalence of ATA in our cohort (30.1%) is in line with the numbers published by others, which have varied between 13% and 36% [2,8,9,12,14,15,17,33,34,36,51]. The patients with ATA in our study were more likely to be male and had higher RSSs (Tables 5 and 6). More common in this patient group were digital ulcers, pulmonary fibrosis, dyspnoea, lung restrictive defect and joint involvement (synovitis, contractures) as well as mouth involvement. However, renal involvement, as measured by proteinuria or renal insufficiency, was not more prevalent in this subgroup, a finding reported by most other researchers [8,9,14,15,33,36,51,52] but not all of them [2,34].

The frequency of RNAP antibodies in our cohort was 3.8%, which is at the lower end of the frequency range reported by others, namely, 10% to 25% in North America [33,52-55], 4% to 31.5% in Europe [14,16,33,35,56-59] and 5% to 11% in Japan [34,36,60]. This may have several reasons: (1) Our cohort is composed of a broad spectrum of SSc patients, including patients with milder forms and with overlap or undifferentiated subtypes of the disease, (2) regional differences due to genetic background and/or environmental influences and (3) different techniques used to ascertain the presence of RNAP antibodies. A high mean RSS, reflecting diffuse skin involvement, was evident for patients with anti-RNAP antibodies (Tables 5 and 6) as previously observed [2,14,33,34,36,40,54-60]. In addition, we found creatine kinase (CK) elevation to be more frequently associated with the presence of anti-RNAP antibodies. This has not been noted before; association with muscular involvement has been found to be nonsignificant [14,34,40,53,54] or even inverse [2,36,55] in previous publications. Our result in this study therefore might be a chance finding due to multiple comparisons. We did not find any significant positive association of RNAP antibodies, or any autoantibody evaluated in this study, with renal involvement. In the German Network for Systemic Scleroderma Registry, 'renal involvement' is defined as renal insufficiency in the form of decreased creatinine clearance and/or proteinuria, as well as a consequence of acute renal crisis. The registry did not include renal crisis as a separate item at that time, which may underestimate the possible correlation of antibodies with renal crisis [18]. Within our network, the frequency of renal crisis currently does not exceed 2% to 3% per year (Hunzelmann N, unpublished observation). The prevalence of renal crisis among patients with RNAP antibodies reported in the literature (for review, see Meyer *et al. *[57]) varies considerably, between 0% and 43%.

Antibodies to fibrillarin (U3-RNP) were most prominent in patients with the diffuse subtype (table 4), which is in accordance with the findings published in previous reports [2,13,34,40,61]. In fact, patients with anti-fibrillarin antibodies, on average, had the highest RSS of all patients in our cohort (Tables 5 and 6). The significance of this finding, however, is less pronounced because of the lower number of patients. The detected anti-fibrillarin antibody frequency of 1.4% was considerably lower than that reported in previous cohorts (2.5% to 19%) [2,13,34,36,40,61-63], which may reflect our broad spectrum of SSc patients that included patients with overlap syndrome and undifferentiated forms, as well as methodological differences and/or the central European background of the patients. Ethnic heterogeneity with a higher frequency of anti-fibrillarin antibodies in black patients has been described previously [2,13,40,61-63]. Our findings of a lower age at disease onset and a higher prevalence of musculoskeletal involvement (Tables 5 and 6) are in line with most previous results [2,36,61,63].

The frequency of PM-Scl antibodies (4.9%) detected in our cohort is in accord with previously published studies in which frequencies between 2% and 6% were noted for SSc patients [1-3,17,33]. These antibodies are most well-known as being typical in patients with dermatomyositis-scleroderma overlap syndrome [1,3]. Accordingly, CK elevation was highly associated with anti-PM-Scl in our cohort. On the other hand, these patients were markedly less likely to have oesophageal involvement (Table 5) and had a low mean ESR (Tables 5 and 6). We found elevated ESR levels in an earlier series of SSc patients with anti-PM-Scl antibodies [32], but others, to the best of our knowledge, did not. (Patients with anti-PM-Scl have rarely been analysed in the large SSc series reported previously.) Therefore, this finding has to be reproduced by independent work in the future. That these patients have a relatively benign prognosis has been mentioned several times before [64-66]. Accordingly, 31% of our patients with anti-PM-Scl antibodies were devoid of any internal organ involvement, compared with 13% of the patients without anti-PM-Scl (*P *= 0.0023, data not shown).

Antibodies to Ku, when found in SSc patients, are often associated with scleroderma overlap syndrome and with muscular involvement [17,36]. Accordingly, we registered a high OR, but with low significance because of the relatively low patient number, for CK elevation associated with anti-Ku antibody (Table 5). The complete absence of ACA and the occasional presence of ATA described in a large previous study that focused on anti-Ku in patients with SSc [17] were nicely reproduced in our cohort (Table 3). Most of our anti-Ku sera (7 of 10) were positive only by IP and negative in a 'classical' precipitation test with native antigen. In a previous study, on the contrary, a similar test (counterimmunoelectrophoresis) was even more sensitive than a line assay to anti-Ku. A possible explanation for this discrepancy might be the source of the antigen, which was of rabbit origin, in our ID assay. Ku autoantibodies are known to tend to be nonreactive with nonhuman antigens [67].

Antibodies to p25/p23 ('anti-chromo') characterise a patient subset within the group of ACA-positive SSc patients. The clinical findings among these patients were heterogeneous in previous reports. Soriano *et al. *[49] found an elevated prevalence of erosive arthritis and Furuta *et al. *[50] reported more interstitial lung disease and liver involvement, whereas Japanese groups [30,31,68] discovered cytopenias, Sjögren's syndrome, overlap with systemic lupus erythematosus and higher ESR levels. We confirmed the relatively strong association with Sjögren's syndrome on the basis of our finding that 20 (71.4%) of 28 patients with p25/p23 antibodies had sicca symptoms, compared to only 41.7% of SSc patients who were negative for these autoantibodies (Table 5). In fact, the weak association of ACA with sicca symptoms (OR = 1.44) (Table 5) lost significance when those patients with co-occurring anti-p25/p23 were eliminated. Likewise, the low mean RSS calculated for ACA-positive patients (6.4) (see Table 6) turned even lower (6.2) after exclusion of patients with anti-p25/p23.

Antibodies to Ro and/or La, as expected and as previously reported [44,45,69,70], were associated with sicca syndrome. This association was only marginally significant; in fact, the antibodies with the most prominent association with the sicca complex were, as mentioned above, anti-p25/p23 antibodies. Anti-Ro and/or anti-La antibodies showed a particularly high correlation with elevated ESR (Tables 5 and 6). An unexpectedly strong association of anti-Ro60 with pulmonary fibrosis (Table 5) was mainly secondary to the relatively high co-occurrence of this antibody with ATA (see Table 3).

No highly significant differences for any of the autoantibody-defined subgroups could be found for gastric, intestinal or renal involvement, including hypertension and reduced renal function. Furthermore, no significant differences dependent on antibodies against aminoacyl-transfer RNA synthetases, To, Sm, SL or NOR-90 were detected, probably because of the low numbers of patients positive for these antibodies. Patients without highly SSc-associated antibodies were more often male and less frequently had Raynaud's phenomenon.

## Conclusions

The occurrence of SSc-related autoantibodies has never been analysed in such detail in a cohort as large as this one. We have shown that five antigens appear to be sufficient to detect more than 95% of the known SSc-associated autoantibody responses in ANA-positive SSc patients. In more than half of patients with a SSc-associated antibody, other nuclear autoantibodies were detected and a considerable patient group (around 40%) still displayed uncharacterised ANAs of as yet unknown significance.

To the best of our knowledge, this is the largest comprehensive analysis of the presence of SSc-associated as well as other non-organ-specific autoantibodies in SSc patients that was performed in a single central laboratory and demonstrates the complexity and heterogeneity of the autoimmune response underlying the pathogenesis of this still enigmatic disease.

## Abbreviations

ACA: anti-centromere antibody; AMA: antimitochondrial antibody; ANA: antinuclear antibody; ATA: anti-topoisomerase I antibody; CK: creatine kinase; ELISA: enzyme-linked immunosorbent assay; ESR: erythrocyte sedimentation rate; HR: hazard ratio; ID: immunodiffusion; IP: immunoprecipitation; OR: odds ratio; RNAP: RNA polymerase; RSS: Rodnan skin score; SSc: systemic sclerosis.

## Competing interests

The authors declare that they have no competing interests.

## Authors' contributions

RM designed the study, performed the serological analyses, had full access to all of the data in the study, analysed the data, takes responsibility for the integrity of the data and the accuracy of the data analysis, interpreted the data, and drafted the manuscript. EG designed the study, was responsible for overall project management, enrolled patients for the network, and contributed data from one participating centre. TK designed the study and was responsible for overall project management. PM, GR, MM, FR, MB, MW, NB, RH, AK, CS, AJ, CP, CF, MS, PL, RS, ESL, CS and IF enrolled patients for the network and contributed the data from the participating centres. IM and UML were responsible for overall project management, enrolled patients for the network and contributed the data from the participating centres. NH interpreted the data and drafted the manuscript, was responsible for overall project management and designed the study. All authors critically revised the manuscript and read and approved the final manuscript for publication.
